# Meglitinides increase the risk of hypoglycemia in diabetic patients with advanced chronic kidney disease: a nationwide, population-based study

**DOI:** 10.18632/oncotarget.17475

**Published:** 2017-04-27

**Authors:** Pei-Chen Wu, Vin-Cent Wu, Cheng-Jui Lin, Chi-Feng Pan, Chih-Yang Chen, Tao-Min Huang, Che-Hsiung Wu, Likwang Chen, Chih-Jen Wu, The NRPB Kidney Consortium

**Affiliations:** ^1^ Division of Nephrology, Department of Internal Medicine, Mackay Memorial Hospital, Taipei, Taiwan; ^2^ Graduate Institute of Clinical Medicine, College of Medicine, National Taiwan University, Taipei, Taiwan; ^3^ Division of Nephrology, Department of Internal Medicine, National Taiwan University Hospital, Taipei, Taiwan; ^4^ Department of Medicine, Mackay Medical College, Taipei, Taiwan; ^5^ Mackay Junior College of Medicine, Nursing and Management, Taipei, Taiwan; ^6^ Department of Internal Medicine, National Taiwan University Hospital Yun-Lin Branch, Douliou, Taiwan; ^7^ Division of Nephrology, Taipei Buddhist Tzu Chi General Hospital, Buddhist Tzu Chi University, Taipei, Taiwan; ^8^ Institute of Population Health Sciences, National Health Research Institutes, Zhunan, Taiwan; ^9^ Graduate Institute of Medical Sciences and Department of Pharmacology, College of Medicine, Taipei Medical University, Taipei, Taiwan; ^10^ Department of Medical Research, China Medical University Hospital, China Medical University, Taichung, Taiwan; ^11^ NRPB, National Research Program for Biopharmaceuticals, ROC, Taiwan

**Keywords:** meglitinide, diabetes mellitus, hypoglycemia, chronic kidney disease, mortality

## Abstract

The safety of short-acting meglitinides in diabetic patients with advanced chronic kidney disease (CKD) has not been widely reported.

Diabetic patients with advanced CKD who had a serum creatinine level of > 6 mg/dL a hematocrit level of ≦ 28% and received erythropoiesis-stimulating agent treatment between 2000 and 2010, were included in this nationwide study in Taiwan.

The outcomes of interest were defined as hypoglycemia and long-term mortality. The risks of hypoglycemia and death were analyzed using Cox proportional hazards models, with end-stage renal disease and anti-diabetic drugs as time-dependent variables.

Fresh users and matched non-users of meglitinides (both *n* = 2,793) were analyzed. The use of meglitinides increased the risk of hypoglycemia (HR, 1.94, p<0.001), as did other anti-diabetic agents. Concomitant use of meglitinide and insuilin will incresase the hypoglycemic risk. (HR, 1.69, p=0.018) Moreover, it was not the use of meglitinides, but the presence of hypoglycemia that predicted mortality. The function curve showed an insignificant trend towards increased hypoglycemic risk in patients aged > 62 and ≤ 33 years from the generalized additive model.

This study suggests that the use of short-acting meglitinides could be associated with increased risk of hypoglycemia in diabetic patients with advanced CKD, especially in patients aged > 62 and ≤ 33 years. Meglitinide combined with insulin will increase hypoglycemia in patients with advanced CKD.

## INTRODUCTION

Diabetes mellitus is a leading cause of death, cardiovascular disease and microvascular complications [1]. Although tighter glycemic control decreases the risk of certain microvascular damage events such as new-onset albuminuria, its effect on reducing death and macrovascular events remain controversial [2–4]. Optimal glycemic control is required to minimize diabetes-associated complications and to avoid the risk of treatment-related hypoglycemia. Drugs that lower postprandial glucose without predisposing patients to hypoglycemia appear to control hyperglycemia and improve cardiovascular outcomes [5].

The challenges for improving glycemic control in patients with chronic kidney disease (CKD) include therapeutic inertia, monitoring difficulties, and the complexity of procurable regimens [6]. The incidence of hypoglycemia is also found to be higher among patients with CKD rather than those without [7]. Altered pharmacokinetics of anti-diabetic agents in CKD patients, decreased renal clearance, peripheral degradation of insulin, and impaired renal gluconeogenesis are all predisposed to the occurrence of hypoglycemia [7].

Meglitinides are short-acting anti-diabetic agents, with a half-life of about one hour, and thus have valuable roles in lowering postprandial hyperglycemia and reducing the danger of hypoglycemia. The main branded drugs in this class are repaglinide and nateglinide. Both repaglinide and nateglinide are metabolized in the liver, and < 10% of repaglinide and most of nateglinide are renally excreted [8, 9]. In clinical practice, meglitinides possess similar potency to metformin and could be an alternative where side effects of metformin are intolerable or where metformin is contraindicated [10], such as advanced CKD. Repaglinide can accumulate in patients with advanced renal dysfunction (eGFR < 30 mL/min/1.73 m2) without a significant increase in hypoglycemia in a small clinical study [11]. Another small-scale study reported that repaglinide was safe and well tolerated in terms of pharmacokinetics and the incidence of hypoglycemia in subjects with varying degrees of renal dysfunction [12]. A metabolite of nateglinide, that has a modest hypoglycemic effect, accumulates in patients with CKD [13].

Experience with the use of meglitinides in advanced CKD is as yet limited; nevertheless, the safety of meglitinides is of particular interest because many advanced CKD patients have been treated with meglitinides for glycemic control in real life [14, 15]. The aim of this study was to examine the risks of meglitinide-associated hypoglycemia and mortality in patients with advanced CKD.

## MATERIALS AND METHODS

### Data source

This population-based cohort study used data retrieved from Taiwan's National Health Insurance (NHI) Research Database, which is one of the largest databases in the world and has been used extensively in various epidemiologic studies [16–19]. The NHI covers almost all 23.7 million people in Taiwan and contains comprehensive healthcare information regarding outpatient visits, hospital admissions, disease profiles, prescriptions, interventional procedures, and vital statuses. All diagnoses are based on the codes of the International Classification of Diseases, 9th Revision (ICD-9).

As all personal information is de-identified in the NHI Research Database, informed consent was waived and this study was exempt from a full ethical review by the institutional review board of the National Taiwan University Hospital (201212021RINC).

### Study population

This study retrospectively included patients aged ≦ 20 years who had diagnosis codes for diabetes mellitus and CKD and received erythropoiesis-stimulating agent (ESA) treatment from January 1, 2000 to December 31, 2010. According to the NHI regulations, ESA can only be prescribed in anemic advanced CKD patients who have a hematocrit level of ≦ 28% and a serum creatinine level of > 6 mg/dL (equivalent to an estimated GFR of < 15 ml/min/1.73 m^2^) with the aim of maintaining a hematocrit level of 33-36%. We only included advanced CKD patients who were fresh users of meglitinides and who had no episode of hypoglycemia within one year prior to the first prescription of ESA. Patients who had been treated with dialysis (identified by procedure codes) or received renal transplantation before ESA and those who did not survive 90 days after the first ESA therapy were excluded.

The baseline comorbidities, including diabetes and CKD, were identified from at least one inpatient claim or three outpatient visits within one year preceding the first ESA treatment. This identification approach has been validated with good predictive power [20–22]. The Charlson comorbidity index was calculated by weighing baseline comorbidities [23].

### Outcome measures

The primary outcome was hypoglycemia (ICD-9 codes: 251.2x), and the secondary outcome was long-term all-cause mortality. Each patient was followed from the date of the first ESA prescription to the date of the hypoglycemic episode, and censored at either death or the end of the study (December 31, 2011), whichever occurred first.

### Exposure assessment

We designed a cohort consisting of fresh users of nateglinide (A10BX03) or repaglinide (A10BX02) after the first dose of ESA, and meglitinide nonusers (defined as one year before enrolling in the study, patients didn't have a history prescription of meglitinides) ). Because studies directly comparing repaglinide and nateglinide are limited, [24–26] in this article we have used the term “meglitindes”, which encompass repaglinide and nateglinide, for the analysis.

**Figure 1:**
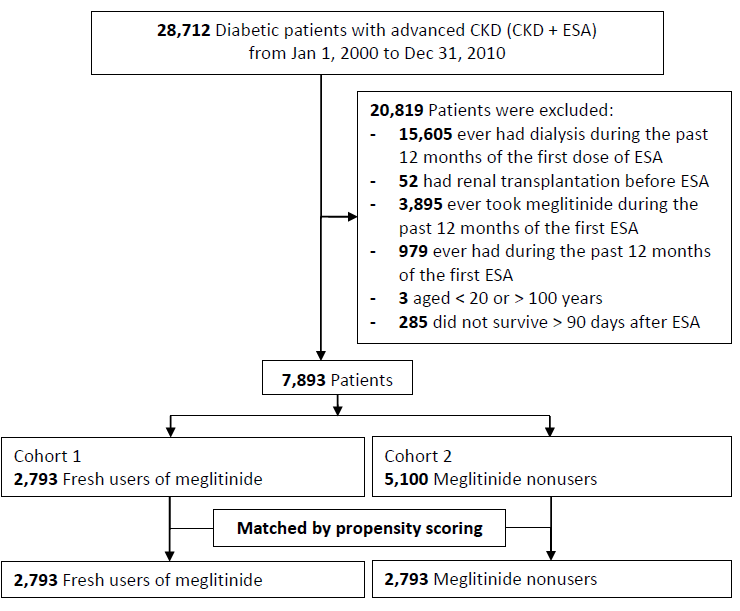
Detailed flowchart for patient enrollment

Fresh users and nonusers of meglitinides were matched at a 1:1 ratio by propensity scoring, with an attempt to make an unbiased estimate of all the confounders predicting meglitinide prescription (Figure 1). After the first dose of ESA, the use of other anti-diabetic agents including sulfonylureas, thiazolidinediones (TZD), dipeptidyl peptidase-4 (DPP-4) inhibitors, and insulin was also recorded. Metformin is not included because it is contraindicated in CKD patients due to the risk of lactic acidosis. Frequency of hemoglobin A1c (HBA1c) measurement within 30 days prior to the event of hypoglycemia or death were also identified. The measurements of HBA1c (09006C) were totally copayed by national health insurance system.

### Statistical analysis

Continuous variables are presented as mean ± standard deviation (SD) or median as appropriate; discrete variables are described as counts and percentages. Independent *t*-tests were used to compare continuous variables and chi-square tests for categorical ones.

Baseline comorbid conditions (listed in Table 1) were inputted into a non-parsimonious multivariable logistic regression model to predict the prescription of meglitinides. The predicted probability derived from the estimated equation was the propensity score for each individual.

**Table 1:** Clinical characteristics and outcomes in patients with advanced CKD, categorized by fresh users and nonusers of meglitinides

	Meglitinide nonusers (*n*=2793)	Meglitinide fresh users (*n*=2793)	*p*-value
**Male gender**	1422 (50.9%)	1432 (51.3%)	0.81
**Age** (year)	63.28±11.65	63.53±11.04	0.83
***Baseline comorbidities***			
Charlson comorbidity index	3.51±1.42	3.48±1.50	0.60
Myocardial infarction	69 (2.5%)	55 (2.0%)	0.24
Congestive heart failure	399 (14.3%)	398 (14.2%)	0.99
Peripheral vascular disease	32 (1.1%)	27 (1.0%)	0.60
Cerebrovascular disease	175 (6.3%)	167 (6.0%)	0.70
Dementia	22 (0.8%)	21 (0.8%)	0.99
COPD	157 (5.6%)	165 (5.9%)	0.69
Rheumatologic disease	15 (0.5%)	14 (0.5%)	0.99
Peptic ulcer	378 (13.5%)	377 (13.5%)	0.99
Hemiplegia	16 (0.6%)	11 (0.4%)	0.44
Malignancy	64 (2.3%)	68 (2.4%)	0.79
Moderate or severe liver disease	109 (3.9%)	132 (4.7%)	0.15
Hypertension	2115 (75.7%)	2109 (75.5%)	0.88
***Anti-diabetic agents***			
Sulfonylurea	1239 (44.4%)	1931 (69.1%)	<0.001
DPP-4 inhibitor	232 (8.3%)	554 (19.8%)	<0.001
Thiazolidinedione (TZD)	393 (14.1%)	836 (29.9%)	<0.001
Insulin	2189 (78.4%)	2481 (88.8%)	<0.001
***Outcomes***			
Hypoglycemia	361 (12.9%)	237 (8.5%)	<0.001
Mortality	1242 (44.5%)	1177 (42.1%)	0.084

Abbreviations: COPD, chronic obstructive pulmonary disease; DPP-4 inhibitor, dipeptidyl peptidase-4 inhibitor

Because all anti-diabetic agents carry the risk of drug-related hypoglycemia and mortality, we evaluated the risk factors of hypoglycemia and death using Cox proportional hazards models [22, 27] with all anti-diabetic agents as well as end-stage renal disease (ESRD) as time-dependent covariates to account for their impacts. In these models, we defined the use of certain anti-diabetic agents as at least one prescription within 30 days prior to the event of hypoglycemia or death. Time-dependent analytical methods have been shown to avoid immortal time bias in observational cohort studies [28, 29]. Variable selection for Cox regression hazards modeling was performed using stepwise multiple regression, with a *p*-to-enter and *p*-to-leave both equal to 0.15 [30]. The final results of multivariate analyses were summarized by hazard ratio (HR) and 95% confidence intervals (CI).

After adjustment for comorbidities, we also conducted an adjusted comparison of risks of meglitinide-associated hypoglycemia among patient subgroups stratified by the status of liver disease and concomitant use of other anti-diabetic agents.

To evaluate the effect of age on the risk of hypoglycemia, we adopted a generalized additive model (GAM) with adjustment for baseline comorbidities and the status of anti-diabetic agent use. This method grants adjustment for possible nonlinear effects of continuous variables [31–33]. The result was shown as a function curve with values of the log odds ratio and was centered to have an average of zero over the range of the data. The approximate point-wise 95% CI was also depicted.

All analyses were carried out using R software, version 3.1.2 (Free Software Foundation, Inc., Boston, MA, U.S.A.). A two-sided *p* value < 0.05 was considered significant.

## RESULTS

### Patient characteristics

Matching of the 2,793 fresh meglitinide users with the same number of meglitinide nonusers as controls was performed using propensity scores (Figure 1). The average age was 63 years, 50% were male, and the Charlson comorbidity index was around 3.5 (Table 1). The proportion of patients with hypertension, cardiovascular disease, moderate or severe liver disease and malignancy were similar in the two groups.

All kinds of anti-diabetic agents were prescribed more frequently in fresh meglitinide users compared to nonusers. In the univariate analysis, the prevalence of hypoglycemia was higher among meglitinide nonusers than among fresh users (*p* < 0.001), but the mortality was not significantly different between the two groups.

### Risk factors for hypoglycemia

In the multivariate time-dependent Cox regression analysis (Table 2), meglitinide, as well as sulfonylurea and insulin, increased the risk of hypoglycemia after risk adjustments (all *p* < 0.001). Age and male gender also slightly increased the hypoglycemic risk, while subsequent ESRD did not. In addition, more frequently measurments of HBA1c, in term of disease status could predict to hypoglycemic events.

**Figure 2:**
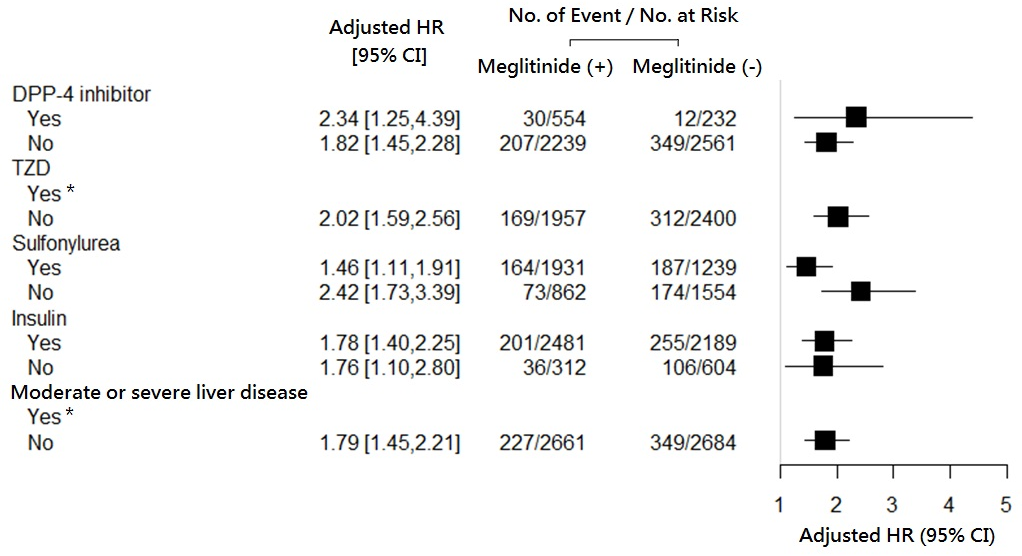
Hazard ratio for fresh use (*versus* nonuse) of meglitinides on the occurrence of hypoglycemia, stratified according to the statuses of concomitant use of other anti-diabetic agents, and of prior liver disease.* Data not shown due to insignificant hazard ratios.

This model exhibited modest discrimination with a c statistic of 0.65 and an adjusted R2 of 0.04. Furthermore, the hypoglycemic risk from meglitinides was consistently elevated across the subgroups stratified by the status of concomitant use of other anti-diabetic agents (Figure 2).

**Figure 3:**
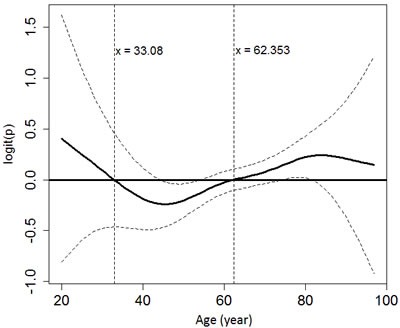
The relationship between age and the probability of developing hypoglycemia using generalized additive modeling and adjusting for gender, end-stage renal disease and the use of anti-diabetic agents

We then evaluated the relationship between age and the risk of hypoglycemia using GAM. The function curve was non-linear and there was an insignificant trend towards increased hypoglycemic risk in patients aged > 62 and ≤ 33 years (Figure 3).

**Table 2:** Risk factors of new onset hypoglycemia by time varying cox regression model^#^

	Hazard Ratio (95% confidence interval)	*p*-value
Age (per year) Male gender Meglitinide* Sulfonylurea* Insulin* ESRD* Frequency of HbA1c measurements*	1.01 (1.00–1.02) 1.17 (1.00–1.38) 1.85 (1.50–2.29) 1.90 (1.58–2.29) 2.08 (1.76–2.45) 0.55 (0.44–0.69) 1.17(1.10-1.24)	0.001 0.053 <0.001 <0.001 <0.001 <0.001 <0.001

Abbreviations: ESRD: End-stage renal disease, HbA1c: Hemoglobin A1c

*, adjusted as time varying model.

^#^, time varying cox regression model, ajudsted with age, gender, comorbidities, antidiabetic agents and Frequency of HBA1c measurement. Table 3: Risk factors of all-cause mortality

### The risk of hypoglycemia on meglitinide from the other agents

We further do the interacting effect of meglitinide and other antidiabetic agents on hypoglycemia. The hypoglycemic effect of meglitinide was consistent among patient subgroups stratified by the status of concomitant use of other antidiabetic agents. However, meglitinide with insulin will increase the risk of hypoglycemia. (*p* = 0.018 ) (Table 4). In addition, most P450 inhibitors will not augment the effect of meglitinide in regarding subsequent hypoglycemi.

**Table 4:** The interaction of medications of interesting to predict new onset hypoglycemia after adding final model*

Co-admission	Interaction with Meglitinide	HR (95% CI)	*p*
***Antihyperglycemic agents***			
DPP4 inhibitor	1.14	0.55-2.34	0.728
TZD	0.64	0.05-2.54	0.224
Sulfonylurea	0.94	0.55-1.59	0.808
Insulin	1.69	1.40-1.98	0.018
***P450 Inhibitors***			
CYF1A2			
Ciprofloxacin	0.35	0.05-2.54	0.301
Amiodarone	1.86	0.81-4.25	0.140
Cimentidine	0.81	0.40-1.68	0.578
CYP2C9			
Fluconazole	0.78	0.11-5.62	0.806
CYP2C19			
Omeprazole	1.18	0.61-2.27	0.619
CYP2A4/5			
Clarithromycin	3.20	0.79-13.03	0.104
Erythromycin	0.56	0.08-4.04	0.568
Ketoconazole	1.03	0.14-7.41	0.975

### Risk factors for all-cause mortality

Table 3 shows the clinical predictors of death after applying the time-varying Cox proportional hazards model. While DPP-4 inhibitor and time-varying ESRD predicted a lower risk of death, factors such as age, male gender, insulin use, hypoglycemia and other baseline comorbidities all predicted mortality. The use of meglitinides had no significant impact on survival (*c* statistic 0.82, adjusted R2 0.55).

**Table 3:** Risk factors of all-cause mortality

	Hazard Ratio (95% confidence interval)	*p*-value
Age (per year) Male gender Hypoglycemia Meglitinides Insulin DPP-4 inhibitor End-stage renal disease (ESRD) Congestive heart failure Myocardial infarction Moderate or severe liver disease Cerebrovascular disease Hemiplegia Rheumatologic disease Hypertension	1.03 (1.03–1.04) 1.11 (1.02–1.20) 13.35 (12.29–14.50) 0.93 (0.82–1.06) 2.22 (2.04–2.40) 0.58 (0.42–0.80) 0.60 (0.54–0.67) 1.47 (1.32–1.64) 1.54 (1.21–1.96) 1.52 (1.27–1.82) 1.17 (1.01–1.36) 2.00 (1.24–3.21) 2.00 (1.13–3.54) 1.11 (1.00–1.23)	<0.001 0.011 <0.001 0.300 <0.001 0.001 <0.001 <0.001 <0.001 <0.001 0.040 0.004 0.017 0.041

Abbreviation: DPP-4 inhibitor, dipeptidyl peptidase-4 inhibitor

## DISCUSSION

This nationwide study, which included a total of 5,586 advanced CKD patients, demonstrated that fresh users of meglitinides had a 1.9-fold greater risk of developing hypoglycemia than meglitinide nonusers. Although short-acting meglitinides are supposed to offer the advantage of having lower incidence of hypoglycemia, our results showed that the magnitude of the risk in advanced CKD patients was comparable to that of sulfonylurea or insulin.

Our advanced diabetic CKD cohort demonstrated an overall prevalence of hypoglycemia (proportion of people) at 10.7%, much higher than that of the overall diabetic population, ranging from 2.4% to 6.3% in two large population-based studies [34, 35]. In the general population, the addition of meglitinide versus placebo to other anti-diabetic agents produced a clinically significant reduction in glycosylated hemoglobin and more frequent but mild hypoglycemia [10]. The main findings for advanced CKD furthermore suggested that meglitinides consistently increased the hypoglycemic risk whether they were or were not combined with other anti-diabetic agents (Figure 2).

Meglitinide-associated hypoglycemia in CKD could be attributed to drug- and non-drug-related factors. Renally excreted meglitinides and metabolites accumulate and have a prolonged half-life in CKD. Uremic toxins further derange glucose homeostasis and insulin metabolism [9]. While augmented hepatic gluconeogenesiss [36], impaired beta-cell response to glucose, and peripheral insulin resistance [37] all predispose CKD patients to hyperglycemia, counteractive mechanisms such as deficient renal gluconeogenesis, impaired renal insulin clearance and degradation, anorexia and diminished glycogen stores, and deficiency in immediate counter-regulatory catecholamine response can render patients as hypoglycemic [7]. The Kidney Disease Outcomes Quality Initiative (KDOQI) guidelines recommend starting nateglinide and repaglinide conservatively at 60 mg and 0.5 mg, respectively, taken with meals for patients with CKD stage 4-5 [38], while other reviews recommend avoiding nateglinide use in patients with CKD stage 5 [9, 39]. Although there are different recommendations, the current body of literature suggests that lower starting doses, prudent monitoring and individualized dose titration are always required to avoid the occurrence of hypoglycemia.

Aging can blunt the autonomic counter-regulatory responses to hypoglycemia and reduces the awareness of low blood glucose condition [40]. Other factors may also contribute to hypoglycemia in the elderly, such as decreased cognition, multiple comorbidities, and polypharmacy [40]. In contrast to previous studies that suggested hypoglycemia is more common in old diabetic patients [40, 41], our results showed an insignificant U-shaped pattern between the risk of hypoglycemia and age in patients with diabetes and CKD. Limited evidence also demonstrated that the events suggestive of hypoglycemia by nateglinide or by glyburide were similar in the elderly and elderly subgroup with renal failure compared with the overall population [42]. More studies are needed to evaluate the interaction between age and CKD in relation to the risk of hypoglycemia.

This study has demonstrated that hypoglycemia, and not meglitinide use, is associated with increased mortality. We showed that meglitinide indirectly could induce long-term mortality rate of by increasing their risk of hypoglycemia. Many antihyperglycemic agents and risk factors will increase the risk of hypoglycemia. The transitive relationship of meglitinide to mortality was taken place by the subsequent hypoglycemia. The concept is in line with meglitinide-related hypoglycemia and other anti-diabetic agent-related hypoglycemia eventually attributing to cardiovascular events, all-cause hospitalization, and all-cause mortality [43]. Hypoglycemia may be associated with augmented sympathetic activity and catecholamine release, leading to confusion, myocardial infarction, and dysrhythmia, which in turn lead to morbidities and mortality.

Our study has a number of limitations. First, the frequency of hypoglycemia might be underestimated. Although the diagnosis codes were regularly scrutinized by the Bureau of NHI and highly accurate, patients with mild or moderate hypoglycemia might be treated at home without seeking medical attention, thereby leading to underreporting of the prevalence of hypoglycemia. Data regarding other factors that might affect the risk of hypoglycemia including the levels of glycosylated hemoglobin, educational status, smoking and alcohol habits, physical activity and body mass index were unavailable from the NHI Research Database. Especially, glycated hemoglobin is tightly associated with risk of hypoglycemia.

However, due to the relatively large sample size, all the errors were likely non-differential and the confounders might not make our results systemically deviated from the truth. Second, our target population of patients with advanced CKD was identified by ICD-9 codes for CKD plus drug codes for ESA. Given that the rate of ESA prescription was 85% in 2012 in predialysis stage 5 CKD patients according to an internal report of the Taiwan Department of Health, we believe the patients on ESA therapy were representative and constituted the majority of stage 5 CKD patients.

In conclusion, this nationwide cohort study provides evidence that fresh use of meglitinides increases the risk of hypoglycemia in predialysis stage 5 CKD patients. As CKD itself increases the risk of hypoglycemia, our results additionally suggest CKD patients taking short-acting meglitinides still require careful monitoring against hypoglycemia. Furthermore, concomitant use of meglitinide and insulin will increase the hypoglycemic risk.
